# Ecological Niche Modeling of Potential West Nile Virus Vector Mosquito Species in Iowa

**DOI:** 10.1673/031.010.11001

**Published:** 2010-07-13

**Authors:** Scott R. Larson, John P. DeGroote, Lyric C. Bartholomay, Ramanathan Sugumaran

**Affiliations:** ^1^ GeoInformatics Training, Research, Education, and Extension Center, Geography Department, University of Northern Iowa, Cedar Falls, IA, USA; ^2^ Department of Entomology, Iowa State University, Ames, IA, USA

**Keywords:** *Aedes vexans*, *Culex pipiens*, *Culex tarsalis*, GARP, Maxent, predictive maps

## Abstract

Ecological niche modeling (ENM) algorithms, Maximum Entropy Species Distribution Modeling (Maxent) and Genetic Algorithm for Rule-set Prediction (GARP), were used to develop models in Iowa for three species of mosquito — two significant, extant West Nile virus (WNV) vectors (*Culex pipiens* L and *Culex tarsalis* Coquillett (Diptera: Culicidae)), and the nuisance mosquito, *Aedes vexans* Meigen (Diptera: Culicidae), a potential WNV bridge vector. Occurrence data for the three mosquito species from a state-wide arbovirus surveillance program were used in combination with climatic and landscape layers. Maxent successfully created more appropriate niche models with greater accuracy than GARP. The three Maxent species' models were combined and the average values were statistically compared to human WNV incidence at the census block group level. The results showed that the Maxent-modeled species' niches averaged together were a useful indicator of WNV human incidence in the state of Iowa. This simple method for creating probability distribution maps proved useful for understanding WNV dynamics and could be applied to the study of other vector-borne diseases.

## Introduction

There is a great need for better planning to control mosquito vectors of existing and emerging viruses, parasitic worms, and protozoa. Ecological niche modeling (ENM) can be used for interpolating and discovering areas of undocumented species' habitats, which in turn could be useful for planners in creating mosquito surveillance programs and mosquito abatement regimens. Iowa has experienced significant human West Nile virus (WNV) incidence and likely serves as a transition zone between WNV vectors in the eastern and western United States (DeGroote et al. 2008). ENM for mosquitoes was uncommon until recently (Levine et al. 2004; Peterson et al. 2005; Benedict et al. 2007; Moffett et al. 2007; Sweeney et al. 2007; Foley et al. 2008) and still has not been utilized for modeling of WNV vector species. This new technology can supplement and help improve existing surveillance programs by describing, in a spatially explicitly way, suitable habitats for mosquito species.

Many ENMs exist including boosted decision trees (BIOCLIM, DOMAIN, and BRT) and various regression models (i.e. GAM, GLM, and MARS). One of the best ENMs, based on a comprehensive review of 17 different methods, is Maximum Entropy Species Distribution Modeling (Maxent), and one of the most commonly used is Genetic Algorithm for Rule-set Prediction (GARP) (Elith et al. 2006). GARP has been utilized extensively at various scales, in different areas of the world, and for various species including plants, animals, and viruses (Peterson et al. 2004; Elith et al. 2006). Recently, many authors have used Maxent to model a variety of species, including birds (Peterson and Papeş 2006), geckos (Pearson et al. 2007), bryophytes (Sérgio et al. 2007), and ticks (Estrada-Peña and Venzal 2007). Both GARP and Maxent require species occurrence records and a set of species-relevant environmental variables in the form of continuous gridded surfaces across the study area. Detailed information exists regarding the parameterization and algorithms in GARP (Stockwell and Noble 1992; Stockwell and Peters 1999) and Maxent modeling (Dudík et al. 2004; Phillips et al. 2004; Phillips et al. 2006).

Although ENM has been effectively used for predicting spatial distributions of mosquito species in other locations, there have been few studies, and none in the Midwest, that spatially predict potential WNV vector species distributions. Two free and commonly utilized ENM programs, openModeller's 1.0.5 implementation of GARP with best subsets and Maxent Version 3.0.6, were used to create and evaluate models of potential mosquito distributions in Iowa.

Previous studies using ENM to model other species have commonly used low resolution datasets and redundant environmental variables with few other types of environmental layers (usually elevation, slope, aspect, land cover, or vegetation cover). Also, ENM is typically run at the scale of a continent, country, or region. For example, Moffet et al. (2007) used 21 environmental variables that contained 8 different layers portraying precipitation and 11 variables related to temperature with a resolution of approximately 4 km2 to study malaria vector species in Africa. Ortega-Huerta et al. (2008) used 46 environmental layers with 29 related to temperature and 13 related to precipitation at a resolution of 18 km2 to model 10 species of birds in Mexico. The present study uses higher resolution data than most, includes more diverse and relevant environmental layers, and encompasses a smaller area than similar ENM studies.

## Materials and Methods

### Mosquito species selection and occurrence data

Mosquito occurrence data, including locations, were acquired from mosquito collection efforts using New Jersey Light Traps placed throughout Iowa. Two important WNV vectors *Culex tarsalis* L and *Culex pipiens* Coquillett (Diptera: Culicidae) (due to morphological similarity, *Culex restuans* Theobald was included with *Cx. pipiens*) were considered, as was *Aedes vexans* Meigen (Diptera: Culicidae). The first two species are likely the most significant transmitters of WNV in Iowa, and *Ae. vexans* is a significant nuisance species with the potential to serve a significant role in WNV transmission as a bridge vector. A bridge vector cannot maintain and amplify WNV in host populations without the help of other species, but only bridge vectors transmit WNV to incidental hosts. These mosquito species were selected based on WNV-vector competence (Turrell et al. 2005) and abundance of the species in Iowa. Because of an interest in recent mosquito population dynamics relating to WNV, only data from 2003–2006 were analyzed in this study. Also, only mosquito occurrences that were spatially unique were incorporated into the GARP and Maxent ENM (many more records exist regarding temporally unique occurrences; however, this study focuses on the spatial distribution of mosquito species). GARP and Maxent also do not allow for more than one occurrence from the same location to be integrated into the modeling process (i.e. a species is either present at a given location or not; the abundance has no effect on the modeling process).

*Cx. tarsalis* is a vector of many pathogens throughout its range (generally, west of the Mississippi River in the United States) including WNV (Goddard et al. 2002; Reisen et al. 2004; Turell et al. 2005), Western Equine Encephalitis virus (Barnett 1956; Reisen et al. 1993; Reisen et al. 1995), and St. Louis Encephalitis virus (Reisen et al. 1993; Reisen et al. 1995). This species regularly has tested positive for WNV in Iowa. From 2002–2006, the percentage of WNV-positive pools was 6.7% (13 out of 193) for *Cx. tarsalis* compared to *Cx. pipiens,* which were found to be positive for WNV 5.2% (41 out of 788) of the time (DeGroote et al. 2008). Larvae occur in varied habitats including roadside ditches, waste lagoons, temporary woodland ponds, marshes, and irrigation water (Edmunds 1955; Rapp 1985). There were 45 spatially unique records for this species; nine spatially unique occurrences of *Cx. tarsalis* were used for validating the Maxent model. While this may seem like a small sample, it is adequate for use in ENM. The effect of predicting species distributions from small numbers of occurrence records has been studied by Pearson et al. (2007), and the results indicated that, from as few as 5 records for Maxent and as few as 10 records for GARP, accurate predictions of presence and absence of a species could be obtained at a success rate of approximately 90% of what is achievable with models based on over 200 records. Other researchers have used ENM for different species with relatively few unique occurrence records. Graham et al. (2004) successfully constructed ENM distributions for several frog species in Ecuador with unique locality records ranging from 6 to 54 per species. Solano and Feria (2007) used ENM to uncover the geographic distribution of species from the genus *Polianthes.* They modeled 12 separate species and three varieties of another species of flowering plants using a range of 3–128 unique localities.

*Cx. pipiens* and *Cx. restuans* were combined into the *Cx. pipiens* group due to difficulties in distinguishing these species based on morphological traits (Darsie and Ward 2005). *Culex pipiens* is an urban species, as its preferred larval habitat is artificial containers such as tin cans, old tires, bird baths, junked cars, etc., but it also readily breeds in storm sewer catch basins, clean and polluted ground pools, ditches, animal waste lagoons, effluent from sewage treatment plants, and other typically eutrophic or polluted water bodies (Kronenwetter-Koepel et al. 2005). It overwinters in the adult stage commonly in crawl spaces under houses. *Cx. restuans* is an earlier season species, and its range reaches north into Iowa. *Cx. restuans* has a similar habitat preference to *Cx. pipiens*: wheel ruts, animal tracks, tires, old cars, and temporary ponds or pools (Siverly 1972). The *Cx. pipiens* group includes major vectors of WNV that routinely test positive for WNV in Iowa, particularly in the eastern part of the state (DeGroote et al. 2008) and in much of North America (Hayes et al. 2005). There were 46 spatially unique records for this species group; nine were used for validation of the Maxent modeling.

Every year, on average, *Ae. vexans* is the single most frequently captured mosquito species in the state of Iowa (Sucaet et al. 2008). According to Siverly (1972), *Ae. vexans* is mostly a floodwater mosquito species, but it can also be found in roadside puddles, woodland pools, vehicle ruts, borrow pits, and waste lagoons. The habitat of *Ae. vexans* includes shaded, sunlit, foul or clean water, and even urban areas. While mainly a nuisance to humans, this mosquito may also play a role as a bridge vector of WNV to humans. Although there is still uncertainty regarding the role of *Ae. vexans* in WNV transmission, findings from numerous studies indicate that the species could potentially serve as a bridge vector to humans and horses and other animals. *Ae. vexans* is certainly associated with humans as a major nuisance mosquito; it is the most common species, on average, in Iowa (Sucaet et al. 2008) and prefers blood feeding from large mammals. Host preferences of *Ae. vexans,* based on blood meal identification, show that it is an opportunistic feeder and even feeds on the American robin (Molaei et al. 2006), an important WNV amplification host in several regions of the USA (Kilpatrick et al. 2006; Molaei et al. 2006). *Aedes vexans* specimens have tested positive for WNV in nature every year in the US from 1999–2008 (CDC, 2009), although far fewer in number than *Cx. pipiens* group mosquitoes. Also, when WNV first arrived in the US in New York City the only two species that researchers found positive for the virus were *Ae. vexans* and *Cx. pipiens* (CDC 1999). *Ae. vexans* can become infected with WNV under laboratory conditions (Turell et al. 2000) and is acknowledged as a competent vector in the laboratory (Goddard et al. 2002; Turell et al. 2005). Research carried out by Tiawsirisup et al. (2008) showed that *Ae. vexans* has the potential to be an enzootic vector involving small mammals (mainly chipmunks). Trevejo and Eidson (2008) conclude from a detailed review of the literature that the principal vectors of WNV in the USA include *Cx. pipiens, Cx. restuans,* and *Cx. tarsalis.* In that review, mosquitoes of secondary importance include *Ae. vexans;* transmission by these secondary vectors is a route by which mammalian hosts can become infected (Trevejo and Eidson 2008). Based on these findings, *Ae. vexans* can be considered a potentially significant bridge vector of WNV In modeling the habitat of *Ae. vexans,* there were 46 unique records used for this species during the study period; nine were used for validation in Maxent.

### Environmental variables

Environmental variables relevant to the species in question were selected based on an assessment of the biology of mosquitoes in Iowa (Table 1). All variables were standardized to a spatial resolution of 360 m^2^ creating grids that were 1542 by 1083 cells (1,120,889 individual cells). This resolution was selected based on a compromise between conserving the information derived from higher resolution data and attaining a reasonable processing speed. The Spatial Analyst extension in ESRI's ArcGIS (www.esri.com) was used to convert all rasters into 360 m2 cell size and then these were converted to ASCII files as required by GARP and Maxent.

The environmental variables fell into two categories: climatic or landscape. The climatic variables included 30-year average annual temperature and precipitation from weather stations throughout Iowa. Climatic surfaces were interpolated from the weather station point data with a minimum curvature spline technique. A surface representing the freezefree period was included and is a categorical variable associated with the number of days without freezing in Iowa. Landscape variables included aspect, slope, compound topographic index (the wetness index, is a function of slope and upstream contributing area), distance to major and minor rivers, land cover, distance to urban areas, available soil water content to a depth of 150 cm, and hydrologic soil groups. Landscape variables on topography, soils, and land cover have been shown to be associated with mosquito populations in numerous studies (Shaman et al. 2002; Diuk-Wasser et al. 2006; DeGroote et al. 2007) and have been generically used for ENM of a variety of species (Anderson et al. 2002; Elith et al. 2006; Stockwell et al. 2006).

Not all environmental layers were used for every species. For example, the distance to the nearest irrigated area layer was only used for *Cx. tarsalis* because larvae commonly exist in irrigated farmland (Edmunds 1955; Rapp 1985) and the species is common in rural areas (Reisen et al. 2006). A built-in jackknifing function in Maxent reduced the environmental layers to only those most important in modeling a single species. This feature rates the usefulness of the environmental layers leading to the rejection of some layers (e.g. a digital elevation model was included in all of the initial ENMs, but it always proved less important and contained less useful information than slope and aspect, two layers derived from the digital elevation model). Also, the *Cx. pipiens* group included urban-centric species, so a layer representing distance to urban areas was used for this species.

### Maxent and GARP modeling

Two different ENM algorithms were used: Maxent and GARP They function in much the same way, both requiring species occurrence records and a set of environmental variables relevant to the studied species (generally temperature, precipitation, vegetation, and elevation) (Anderson et al. 2002; Tsoar et al. 2007). For Maxent, 20% of the occurrence records were set aside for external validation, and the maximum number of iterations was set at 1000. The occurrence records that were set aside for validation were chosen at random by Maxent. The remaining 80% of the records were used in the construction of the Maxent niche models. Jackknife tests in Maxent were used to limit the number of environmental layers to only those layers that showed a substantial influence on the distribution of the mosquito species (Table 1). Using the same environmental variables, a model was created using GARP with best subsets - the new openModeller implementation. All of the occurrence records for each species were included in the construction of the GARP models. The method in which GARP constructs a model is quite different from other algorithms. GARP creates a set of rules that predict the ecological niche of a given species. However, in this process of model building, only 50% of the occurrence points are used in the construction of any single given rule. The other 50% are then used to validate the legitimacy of this one rule. Then GARP decides (based on predictive power) whether the rule should be included or excluded from the final set of rules. After this, GARP uses 50% of the occurrence points (again chosen at random) to construct the next rule in the series. In this manner, the validation dataset is the exact same as the dataset used to create the model. The parameters used in the GARP model were left at the default values except for total runs which were increased from 10 to 50. Default values included using 50% of the occurrence records for training and 50% for validation (see above). The number of threads can be specified if analyses are run on a computer with multiple processors. The other values have little effect on the final outcome of the analysis but will affect the processing time of the analysis. Changing certain parameter's values will stop the analysis early, and manipulating certain values will lengthen the time needed for model construction. Given that the number of environmental layers used and the number of occurrence records were relatively few in these ENM analyses, using more conservative input parameters did not increase the stability of the models but caused significant increases in computing time.

**Table 1.**
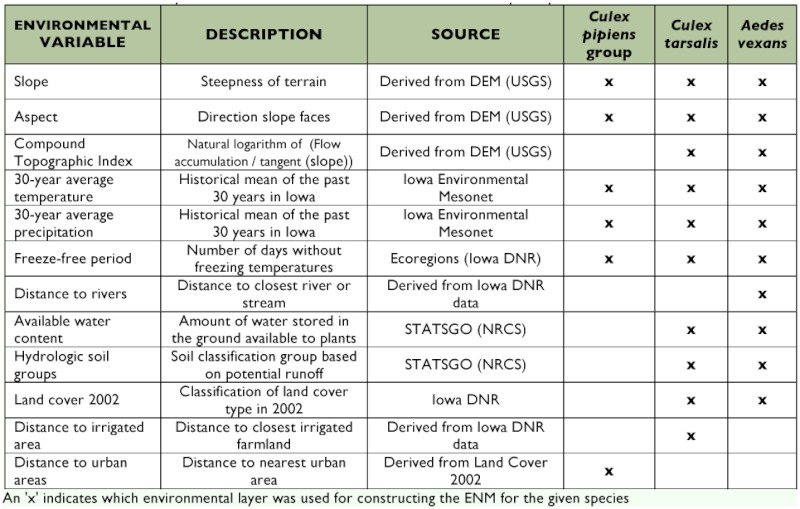
Environmental layers used in the construction of ENM for three mosquito species in Iowa

### Evaluation: Comparison to human WNV incidence

The individual models were compared to human WNV cases, but no significant correlation was found between any of the GARP or Maxent models for the three species. In order to uncover a connection between mosquito habitats and human WNV cases, the individual Maxent and GARP models for the three species were combined and averaged into a single raster dataset for comparison to human WNV incidence data. Geocoded human WNV incidence data were aggregated to census block groups provided by the Iowa Department of Public Health for the years 2002–2006 as described in DeGroote et al. (2008). Zonal statistics functions in ArcGIS were used to compile the average scores from the averaged ENMs by census block group. Bonferroni multiple comparison procedures (Ott and Longnecker 2006) were utilized to examine the relationship between the number of WNV cases and averaged ENM values.

## Results and Discussion

### Ecological niche modeling for individual mosquito species

**Figure 1.**
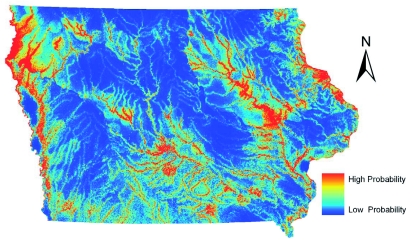
ENM for *Culex tarsalis* in Iowa using Maxent. High quality figures are available online.

**Figure 2.**
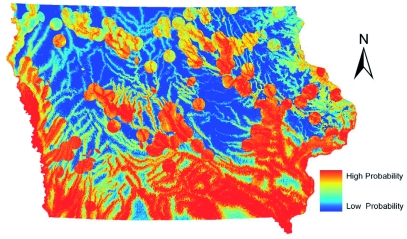
ENM for *Culex tarsalis* in Iowa using GARP. High quality figures are available online.

The Maxent-created model for *Cx. tarsalis* (Figure 1) highlighted the northwestern area of Iowa as an area likely to be *Cx. tarsalis* habitat. Irrigation is commonly employed in this area to grow row crops. This model is in accordance with the biology of this mosquito, because *Cx. tarsalis* are frequently associated with irrigated cropland (Surtees 1970). The models for *Cx. tarsalis* included a layer on the distance to the nearest irrigated farmland to include these important habitable areas in the models. *Cx. tarsalis* is considered an enzootic vector and most likely is a bridge vector of WNV to humans in Iowa, and the northwest area of the state is the most common area in Iowa for human WNV cases (DeGroote et al. 2008). Also prevalent in the Maxent model was the distance to rivers layer. The GARP model (Figure 2) differed quite drastically for the predicted habitat of *Cx. tarsalis.* The distance to irrigated areas layer used in the construction of the GARP model created a pattern of cells with at least two areas that have been allowed and given permits by the state of Iowa to use irrigated water within 10 km. Sixty-three percent of unique occurrence points met this condition, and those influenced the modeling strongly. Also noteworthy was a surprising section of southern Iowa that was predicted to be habitable. Lower numbers of *Cx. tarsalis* are collected in this area, compared to the rest of the state (Sucaet et al. 2008), suggesting that this is an example of GARP overpredicting the niche of a species, as has been seen in other studies (Peterson et al. 2002; Phillips et al. 2004; Elith et al. 2006; Sánchez-Flores et al. 2007; Yun-sheng et al. 2007). Visual inspection of the environmental layers used in the construction of the ENM for *Cx. tarsalis* revealed that a combination of layers including higher average temperatures combined with various other layers including distance to rivers, soil properties, and grassland cover was likely responsible for this possible exaggeration of predicted habitat in the southern one-third of the state.

**Figure 3.**
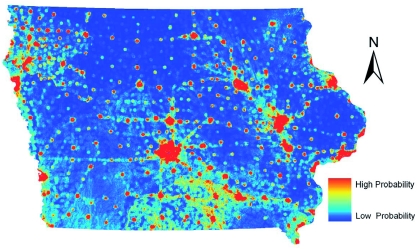
ENM for *Culex pipiens* in Iowa using Maxent. High quality figures are available online.

**Figure 4.**
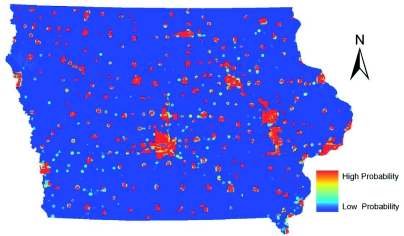
ENM for *Culex pipiens* in Iowa using GARP. High quality figures are available online.

Models created for the distribution of *Cx. pipiens* group are shown in Figures 3 and 4. *Cx. pipiens* is considered an urban species, and most of the predicted habitat in both models was within or near residential and commercial areas. The GARP model more strictly predicted habitat areas in or very near urban areas. Approximately 89% of occurrence records fell inside or within four km of an incorporated city boundary. Based on a visual comparison between the environmental layers and the GARP model, the GARP model appeared dominated by the distance to urban areas layer with other layers having limited influence. Maxent predicted a greater area with low to moderate probabilities across the state and higher probabilities near urban areas. The Maxent model seemed to be influenced by the other environmental layers much more than the GARP model. A greater area in the southern part of the state was probably influenced by the climatic layers. Maxent and GARP seemed to model some linear features (i.e. roads). This may have been an artifact of misclassified road pixels in the land cover data that were wrongly classified as commercial/industrial.

**Figure 5.**
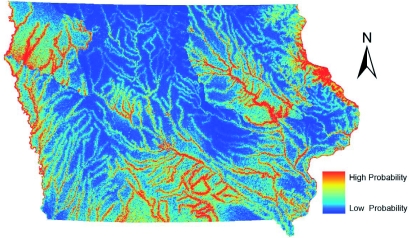
ENM for *Aedes vexans* in Iowa using Maxent. High quality figures are available online.

**Figure 6.**
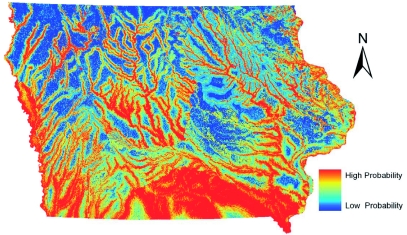
ENM for *Aedes vexans* in Iowa using GARP. High quality figures are available online.

The two models created for *Ae. vexans* (Figures 5, 6) are dominated by the distance to rivers layer, based on visual analysis. Approximately 90% of the *Ae. vexans* presence points fell within 4 km of a major river in Iowa, and the models appropriately showed likely habitat in potential floodplains. However, the GARP model predicted a greater area of Iowa as probable habitat, especially in southern Iowa. It is likely that the GARP model overpredicted the fundamental niche of this species also. In southern Iowa, the GARP model seemed to be heavily influenced by the grassland areas from the land cover data. This was based on comparing the model to the land cover data visually. Due to the sampling regime, many points fell in mapped grassland cells that were in a mixed landscape fabric around cities (possibly misclassified cells). Subsequently, the model likely overpredicted the habitat of *Ae. vexans* in the grassland dominated areas of southern Iowa. In an eastern Iowa county, DeGroote et al. (2007) showed a weakly positive correlation between *Ae. vexans* counts and grassland areas, while showing a much stronger positive correlation to forested areas, which generally fall along river corridors in Iowa.

**Figure 7.**
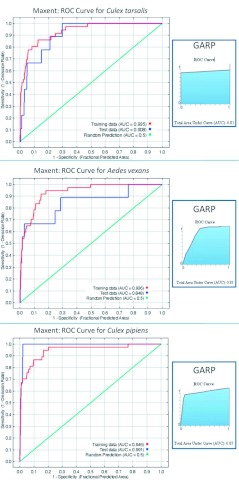
ROC curves and AUC values for all Maxent and GARP models. High quality figures are available online.

Receiver operating characteristic (ROC) curves and the area under the curve (AUC) values were used to compare the models constructed using GARP and Maxent for each of the species being studied. An AUC score of one would mean perfect prediction with zero omission (an AUC score equal to 0.5 would be expected from a random prediction). This is a standard method for analyzing ENM (Phillips et al. 2004; Elith et al. 2006; Sérgio et al. 2006), and the AUC can be a useful indicator of accuracy between ENM models. See Figure 7 for the ROC curves from both Maxent and GARP models and AUC values for each of the species' models. GARP produces only one ROC curve and AUC value due to the nature of the algorithm (see above), but Maxent produces two ROC curves (based on either the initial (training) data and on the validation (test) data) with associated AUC values. The AUC values based on the validation dataset for the Maxent modeling were 0.848, 0.908, and 0.991 for *Ae. vexans, Cx. tarsalis,* and the *Cx. pipiens* group, respectively. However, the AUC values from the Maxent models in relation to the initial (training) datasets were 0.936 for *Ae. vexans,* 0.935 for *Cx. tarsalis,* and 0.946 for *Cx. pipiens.* The AUC values derived from the GARP models were 0.81 for *Ae. vexans,* 0.81 for *Cx. tarsalis,* and 0.87 for *Cx. pipiens.*

### Comparison to human WNV incidence

**Figure 8.**
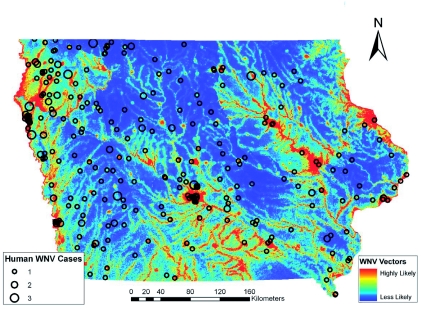
Averaged mosquito distributions of the three Maxent models overlaid with West Nile virus cases based on census block group centroids. High quality figures are available online.

Initially, the individual habitat maps were compared to human WNV cases, but no single species' predicted habitats compared favorably. WNV transmission to humans is complicated by the existence of both enzootic and bridge vector species. Since both types of vectors are needed for incidental hosts (i.e. humans) to become infected, it was decided to combine the predicted habitats for *Cx. pipiens, Cx. tarsalis,* and *Ae. vexans.* After experimenting with weighting the different species based on estimated transmission rates or abundance of mosquito species, the probabilities of these species occurrences were averaged in order to define areas that have both enzootic and bridge vector species (a criterion for transmission of WNV to humans). This shared habitat was then compared to human WNV cases in Iowa at the census block group level. Figure 8 shows the raster surface created by averaging the individual Maxent probability distribution models overlaid with human WNV incidence, symbolized by graduated symbols based on the census block group centroid. The combination of the probability distribution models highlights the river systems in Iowa. Iowa is considered one of the most ecologically disrupted states. Based on surveys conducted in the mid-1800s, the landscape of Iowa was dominated by prairie that occupied 28.5 million acres (79%) of the state; 99.9% of those acres have been converted to agricultural land (Smith 1998). Therefore, much of the suitable habitat for mosquito species that do not normally seek out agricultural areas or urban environments is likely to occur along the streams and rivers with their associated boundary forests and floodplains with wetland-like corridors. The Bonferroni multiple comparisons procedure showed that there were statistically significant variations in mean values of combined model scores in census block groups with zero (0.097058), one (0.0886), two (0.099844), and three (0.152527) cases of WNV in humans. The average value for the cells that fell into census block groups with only one case of WNV was actually lower than the average of the values of cells that fell into census block groups with zero cases of WNV However, the average values of cells that fell into census block groups with either two or three cases of WNV were significantly greater (p < 0.05) than the average value of cells that fell into the census block group with either zero or one WNV case. A person's residence is not necessarily the site of virus transmission. However, when multiple cases of WNV in humans occur in the same census block group, it is more likely that a ‘hot spot’ for virus activity exists in that area, and thus this analysis indicates that the combined Maxent models highlight areas of higher risk (DeGroote et al. 2008). Individual species predicted habitats compared to human WNV cases resulted in no statistically significant differentiation between census block groups. The combined GARP models failed to show significant differences between census block groups with varying numbers of WNV cases.

## Conclusions

In conclusion, these probability distribution maps are an initial step in understanding the transmission of mosquito-borne pathogens in the state of Iowa, a probable transition zone between WNV vectors in the eastern and western parts of the USA and a common site of human WNV infection. Maxent appears to be better able to fit the occurrences of mosquito species without overpredicting the area in which they are able to live, a common drawback of GARP (Peterson et al. 2002; Phillips et al. 2004; Elith et al. 2006; Sánchez-Flores et al. 2007; Yun-sheng et al. 2007). Census block groups with greater numbers of human WNV cases had higher average probability scores for the combined Maxent models for the three species. This indicates that this methodology is valuable in creating a static WNV human risk map for the whole state based on ecologically relevant data.

Ideally, a more exhaustive sampling regime would allow for mosquito traps to be placed in a continuous grid throughout the state. In reality, the sampling regime is restricted by resource and logistical constraints, but is datarich in that the same sites have been sampled for many consecutive years. Using this sampling regime, ENM has proven to be a useful method for determining the overall distribution of different mosquito species in the state of Iowa, which is 145,743 km2 in size. Ecological niche modeling is useful for interpolating the distribution of mosquitoes in unsampled and undersampled areas. The probability maps created for this study can help to inform researchers where to place other types of mosquito surveillance equipment such as gravid traps and CDC-style CO2-baited mosquito traps, which collect live samples valuable for testing mosquitoes for WNV Ground truthing in undersampled sites would provide additional validation of the models developed herein.

The next step would be to use ENM with climatic data (i.e. precipitation, temperature, humidity, etc.) or remotely sensed derived data such as the Normalized Difference Vegetation Index, which can be used as a surrogate for climatic data, for different time periods in order to discover not just the spatial distribution of vectors but also their temporal population dynamics. This could help to inform further efforts to predict, in near real time, the distributions of potential WNV vectors which pose a health risk to humans across the state of Iowa.

## Abbreviations

AUC: area under the curve;
ENM: Ecological niche modeling;
WNV: West Nile virus;
GARP: Genetic Algorithm for Rule-set Prediction;
Maxent: Maximum Entropy Species Distribution Modeling;
ROC: Receiver operating characteristic
